# Development of a Fast and Efficient Strategy Based on Nanomagnetic Materials to Remove Polystyrene Spheres from the Aquatic Environment

**DOI:** 10.3390/molecules29194565

**Published:** 2024-09-25

**Authors:** Yésica Vicente-Martínez, Irene Soler-García, Manuel Hernández-Córdoba, Ignacio López-García, Rosa Penalver

**Affiliations:** Department of Analytical Chemistry, Faculty of Chemistry, Regional Campus of International Excellence “Campus Mare Nostrum”, University of Murcia, 30100 Murcia, Spain; yesicavm@um.es (Y.V.-M.); irene.solerg@um.es (I.S.-G.); hcordoba@um.es (M.H.-C.); ilg@um.es (I.L.-G.)

**Keywords:** remediation, microplastics, water pollution, micro-polystyrene, magnetic nanosorbents

## Abstract

Microplastics contamination is growing globally, being a risk for different environmental compartments including animals and humans. At present, some Spanish beaches and coasts have been affected by discharges of these pollutants, which have caused a serious environmental problem. Therefore, efficient strategies to remove microplastics (MPs) from environmental samples are needed. In this study, the application of three magnetic materials, namely iron oxide (Fe_3_O_4_) and the composites Fe_3_O_4_@Ag and Fe_3_O_4_@Ag@L-Cysteine, to remove MPs, specifically polystyrene (PS), from water samples has been assessed. The magnetic nanoparticles were synthesized and characterized by field effect scanning electron microscopy with energy dispersive X-ray spectroscopy detection (FESEM-EDX). Experimental conditions such as temperature, time, and pH during the removal process were assessed for the different adsorbent materials. The removal rate was calculated by filtering the treated water samples and counting the remaining MPs in the water using ImageJ software. The strongest removal efficiency (100%) was shown using Fe_3_O_4_@Ag@L-Cysteine for PS at 50 mg L^−1^ within 15 min of the separation process at room temperature and a neutral pH. A thermodynamic study demonstrated that the developed MPs elimination strategy was a spontaneous and physisorption process. Coated Fe_3_O_4_ magnetic nanoparticles were demonstrated to be an efficient adsorbent for MP removal in aquatic environments and their use a promising technique for the control of MPs contamination.

## 1. Introduction

Plastic production is one of the main sources of environmental pollution. Due to its low degradability, plastic waste is present in the environment for a long time, producing a risk to the ecosystem including all living organisms [1].

Nowadays, small plastic debris (≤5 mm) known as microplastics (MPs) [2] have become significant emerging pollutants due to their properties such as ubiquity, small size, high persistence, and complexity [3]. These contaminants cause negative effects not only on the environment but also on human health, generating global concern [4,5].

MPs are commonly detected in aquatic ecosystems and their negative effect on marine organisms has been demonstrated [6,7,8], making this environmental compartment the focus of research. Several sources of MPs in the marine environment have been described in the literature [6,9] highlighting land-based activities including leaching from landfills, coastal tourism, riverine import, and atmospheric relocation, as well as wastewater treatment plants (WWTPs) [10]. Once in the marine environment, MPs can be incorporated into the food chain through ingestion by the various organisms that live in that habitat [11]. Ingestion is the most common way of incorporation of MPs into humans and several negative effects of these pollutants on human health have been demonstrated including oxidative stress, metabolic disorder, neurotoxicity, and immune response, as well as developmental and reproductive toxicity [12].

Among the different polymeric materials, polystyrene (PS) is one of the most used for packaging, medical items, consumer electronics products, construction, and agriculture due to its colorlessness, transparency, and thermally insulating characteristics [13,14]. Due to its extensive use, polystyrene-microplastic (PS-MPs) is a pollutant present in different environments, including aquatic, terrestrial, and atmospheric systems [15,16,17]. Therefore, their ubiquity and demonstrated toxicity in marine animal tissues [18,19] make PS-MPs of special interest for researchers.

There is a clear urgent need to develop strategies that allow the removal of these pollutants from the environment, considering their negative impact on the ecosystem and human health by incorporation into the food chain [20,21,22]. Specifically, the limitations in removing MPs in a fast and efficient way have been recently demonstrated on the coast of Galicia (Spain), which was affected by discharges of these pollutants. MP removal methodologies may be classified into chemical, physical, and biological approaches [23].

Physical remediation includes all separation methodologies used in waste-water treatment [23] involving the traditional separation methods such as those applying grids, flotation, sedimentation, and filtration [24]. Overall, these procedures are efficient and simple but limited to a certain volume of sample and to potential airborne MPs contamination [25]. In chemical strategies, synthetic compounds are applied for flocculation and coagulation, which are the most common processes to remove MPs especially in WWTPs [26]. Chemical coagulation is a simple and fast process, but a high amount of coagulant is normally needed. Additionally, Shi et al. [27] defined chemical methodologies using polymer oxidation-related processes. These oxidation steps allow in-situ polymer degradation; however, the operation cost is usually high. To overcome those limitations, biological treatments based on the use of microorganisms to degrade MPs from the environment are currently growing; however, this promising approach usually takes a long time [23].

Current studies are focused on the development of innovative treatment techniques to remove MPs from the environment. The application of new nano-adsorbents materials in this field is of special attention to researchers, considering the large surface areas per unit mass, which provides a high global density of adsorption sites [28,29]. In addition, technology based on magnetic nanoparticle (NP) adsorbents is growing due to their potentially high efficiency and non-negative environmental impact since these particles may be removed by magnetism without incorporation into the environment. 

Magnetic nano-Fe_3_O_4_ adsorbents have been applied to remove MPs from marine environments with a removal efficiency between 62 and 83.6% [27]. Heo et al. [29] found higher extraction efficiencies applying Fe_3_O_4_ NPs as an adsorbent to remove specifically polystyrene-microplastics (PS-MPs). High removal efficiencies were also obtained by Tang et al. [30] using magnetic carbon nanotubes as an adsorbent, but long interaction times (5 h) were required.

However, Fe_3_O_4_ NPs have high reactivity and may suffer degradation in specific environments. Therefore, functionalization of the NP surface by organic or inorganic components may improve the biocompatibility and dispersibility of the NPs facilitating their usage and reutilization [31]. Fe_3_O_4_ NPs coated with hexadecyltrimethoxysilane have been tested, showing promising removal efficiencies from 78% to 93% depending on the type of MPs and their size. Some limitations were found in that study due to the fragmentation of the more brittle MPs during the removal process, potentially due to the strength of the magnet [32]. Polymeric coatings have been also tested, specifically polydimethylsiloxane (PDMS), for MP removal, demonstrating extraction efficiencies around 90% depending on the MP [33]. Adsorption mechanisms were evaluated using ball-milled magnetic pinewood biochar for removal of PS and functionalized PS. The three different pinewood biochars used by the authors were prepared at three different pyrolysis temperatures and then were magnetized with Fe_3_O_4_ by mixing them with agate balls of different sizes achieving a removal efficiency of 82.73% [34]. The synthesis of the abovementioned functionalized NPs requires high temperatures [34] or several extraction steps [33], unlike the NPs selected in this study (Fe_3_O_4_@Ag@L-Cysteine) for which synthesis does not involve any extraction or heating step making the proposed procedure simple and low-cost.

In the present work, a solution to the serious problem of microplastics contamination in water has been studied. Specifically, the removal efficiencies of PS-MPs from water samples by different magnetic Fe_3_O_4_ NPs (Fe_3_O_4_@Ag and Fe_3_O_4_@Ag@L-Cysteine) were compared. PS-MPs were used in this study as a model due to their high contribution to MP pollution in the different environments as described above. The adsorption mechanism between the synthesized magnetic NPs and PS-MPs was elucidated through an exhaustive study of the pH for each adsorption process and the potential of zero charge (PZ0) for each species involved, providing relevant information for the investigation of adsorbent materials for removal strategies.

The methodology presented in this work allowed higher removal efficiency (100%) within shorter contact times (15 min) than previous studies, highlighting the absence of heating during the removal process when Fe_3_O_4_@Ag@L-Cysteine NPs were used. The thermodynamic study of the process by calculating the Gibbs free energies showed that in all cases there was physisorption and the procedure was spontaneous.

Moreover, adsorbents were characterized by field effect scanning electron microscopy and energy dispersive X-ray before and after the adsorption process. The water samples were filtered and the number of PS-MPs in the initial and treated samples was measured using ImageJ software to calculate the removal efficiency. Additionally, it is worth mentioning that the developed strategy is environmentally friendly since the NPs are removed from the medium, using a magnet, to be reutilized. Altogether, this work presents a simple and novel strategy to remove MPs from water samples. Therefore, this methodology is presented as an efficient alternative to conventional WWTPs, where the removal efficiency of MPs is limited due to several factors such as the shape and size of the MPs, the hydraulic retention time, or the overloading of the WWTP. Therefore, the use of innovative technologies like the one presented in this work is highly necessary to ensure efficient treatment of wastewater [25,26].

## 2. Results and Discussion

### 2.1. Characterization of Fe_3_O_4_, Fe_3_O_4_@Ag, and Fe_3_O_4_@Ag@L-Cys before PS-MPs Removal

The adsorbents used in this study were characterized before being used in the adsorption process by field effect scanning electron microscopy (FESEM) and energy dispersive X-ray microscopy (EDX).

**Figure 1:**
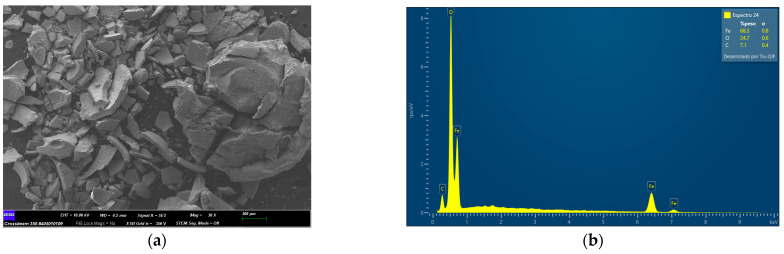
(**a**) FESEM image at 30× magnification of Fe_3_O_4_ before the removal process; (**b**) EDX spectrum corresponding with the whole area of (**a**).

Figure 1a shows the FESEM image for Fe_3_O_4_. In Figure 1b, the EDX spectrum corresponding to the whole area of Figure 1a image can be seen.

**Figure 2:**
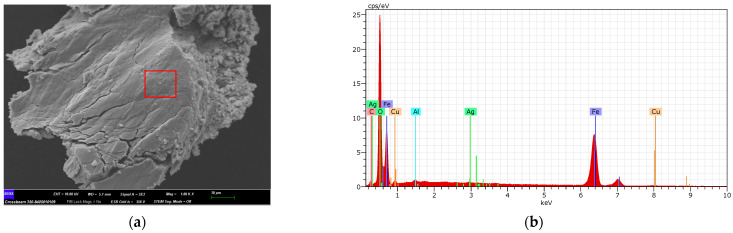
(**a**) FESEM image at 1000× magnification of Fe_3_O_4_@Ag before the removal process; (**b**) EDX spectrum for the area marked in red in (**a**), where signals corresponding to Ag atoms can be seen.

**Figure 3:**
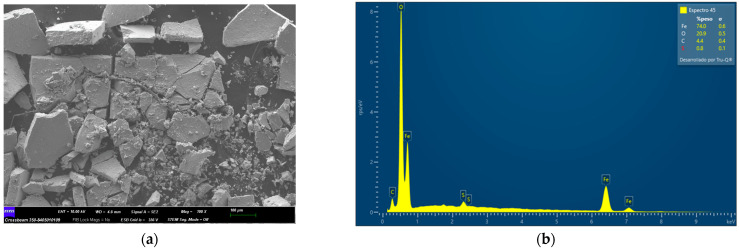
(**a**) FESEM image at 100× magnification of Fe_3_O_4_@Ag@L-Cys; (**b**) EDX spectrum corresponding to the whole area of (**a**). As can be seen, signals corresponding to S atoms from Cysteine appear in the spectrum.

In Figure 2a, there are some bright spots corresponding to the silver atoms that cover the ferrite nucleus (red box), as some authors have indicated previously [35]. The EDX spectrum corresponding to the area marked in red in Figure 2a can be seen in Figure 2b, where the silver atoms signals appear with respect to the adsorbent shown in Figure 1. Moreover, Figure 3b shows the EDX spectrum of Fe_3_O_4_@Ag@L-Cys corresponding to the whole area of the FESEM image shown in Figure 3a. Clear signals of S, C, and N atoms coming from L-Cys can be seen.

**Figure 4:**
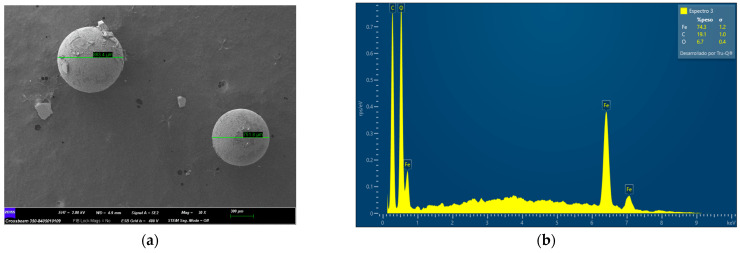
(**a**) FESEM images at 30× magnification of Fe_3_O_4_ after PS removal, displaying larger spheres; (**b**) EDX spectrum corresponding to whole area; here, signals corresponding to C atoms from PS-MPs appear.

Furthermore, after the PS-MP removal process, the adsorbents were again characterized by means of FESEM. Figure 4a shows polystyrene microspheres on the adsorbent Fe_3_O_4_ and the corresponding EDX spectrum of the sample is shown in Figure 4b. The appearance in this spectrum of signals corresponding to C atoms as well as Fe and O atoms shows the presence of polystyrene on the magnetic ferrite.

### 2.2. Effect of Contact Time on PS-MP Removal

**Figure 5:**
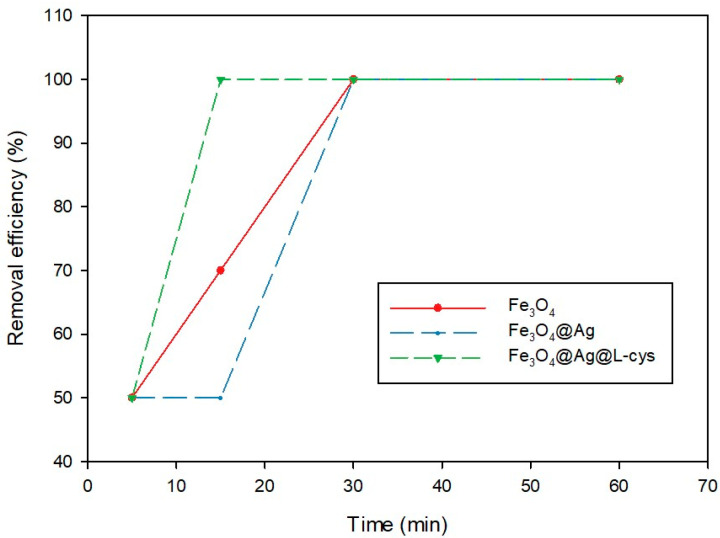
Dependence of the contact time between the magnetic adsorbents and the suspension containing 50 mg L^−1^ of PS-MPs versus the removal efficiency.

For each type of adsorbent, the time necessary for the complete separation of PS-MPs was studied. For this purpose, 500 μL of each adsorbent suspension was employed and the removal efficiency was studied at 5, 15, 30, and 60 min. The aqueous solution containing 50 mg L^−1^ of PS-MPs corresponds to two or three PS spheres in a 10 mL water sample; each adsorbent was kept under orbital agitation at the times indicated above. The results are summarized in Table 1 and are plotted in Figure 5.

As can be seen in Figure 5, when the adsorbent used was Fe_3_O_4_@Ag@L-Cys, the procedure took only 15 min to achieve total PS-MP removal, whereas when the adsorbents employed were Fe_3_O_4_ and Fe_3_O_4_@Ag, 30 min under agitation was required to reach 100% removal efficiency.

### 2.3. Effect of Adsorbent Dose on PS-MP Removal Efficiency

**Figure 6:**
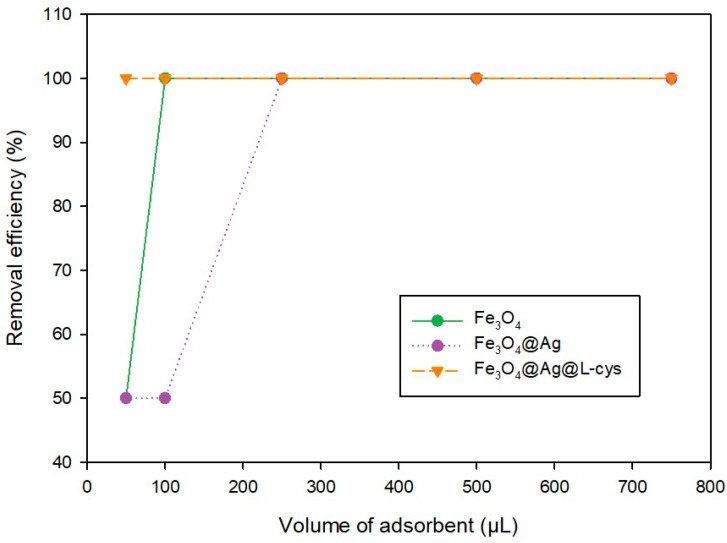
Dependence of the dose of each adsorbent on the removal efficiency of the process.

The optimal dose of adsorbent to carry out a complete separation of PS-MPs from water solution was studied. Water samples containing PS-MPs at concentration of 50 mg L^−1^ were treated with different volumes of each adsorbent suspension for 30 min, namely 50, 100, 250, 500, and 750 μL. As shown in Figure 6, when the adsorbent used was Fe_3_O_4_@Ag@L-Cys, only 50 μL was necessary to reach 100% efficiency removal. However, when the adsorbent employed was Fe_3_O_4_@Ag, 250 μL was necessary to achieve the total removal of polystyrene from water. An intermediate situation was achieved when the elimination process was carried out using Fe_3_O_4_ as adsorbent, since in this case 100 μL of the corresponding suspension were needed to eliminate completely PS-MPs from the water.

### 2.4. Study of the Effect of the pH of the Aqueous Solution Containing PS-MPs on the Removal Efficiency and Adsorption Mechanism

**Figure 7:**
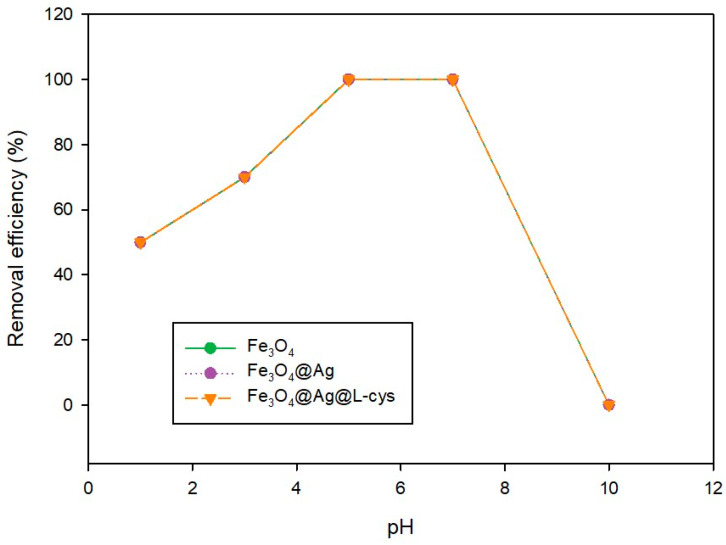
Dependence of the pH of the media on the removal efficiency of the process for each magnetic adsorbent.

A study of the pH of the aqueous medium was carried out to assess the optimal value of pH to achieve the greatest PS-MP (50 mg L^−1^) removal efficiency. For this purpose, the process described above was carried out at the following pH values: 1, 3, 5, 7, and 10. As shown in Figure 7, at pH = 5 and pH = 7 the total elimination of PS-MPs was achieved using any of the three adsorbents studied. When the pH was acid (1 and 3) the elimination was partial and at pH = 10 no PS-MPs were eliminated. Therefore, neutral pH was selected as the optimum pH value for polystyrene removal with the three adsorbents.

To elucidate the possible removal mechanism of PS-MPs with the adsorbents tested, it is necessary to take into account the PZ0 of each adsorbent and adsorbate, as well as to study the relationship of these values with the removal efficiencies achieved at each pH measured. As indicated by several authors, from a pH value between 5 and 7 to basic pH, the surface of the polystyrene microspheres gradually acquires a negative charge [36]. While at more acidic pH values, polystyrene is positively charged [37]. At pH > 7, a repulsion phenomenon appears between the pollutant and the adsorbents, since the surface of the adsorbents would also be negatively charged (PZ0 Fe_3_O_4_ = 6.0, PZ0 Fe_3_O_4_@Ag= 6.7, and PZ0 Fe_3_O_4_@Ag@L-Cys = 5.8) [38]. Below pH 5, the adsorbents and the polystyrene are positively charged, so the repulsion phenomenon between adsorbent and adsorbate appears again, making the removal process more inefficient. At pH = 5, the PS-MPs start to become negatively charged and the adsorbents are positively charged, so the attraction between both is evident and the removal is total. This attraction is maintained up to neutral pH because from pH = 5 to neutral pH = 7 the negative charge of the adsorbents is not very high and neither is that of the polystyrene, so another type of non-electrostatic interaction is taking place so that the removal efficiency reaches values of 100%. As explained in the next section, the calculation of the Gibbs free energy of the adsorption process of PS-MPs on the adsorbents indicates that it is physical adsorption. However, the values of ΔG^0^ are very close to the limit where there is a mixed chemo-physisorption process for Fe_3_O_4_ and Fe_3_O_4_@Ag, so it is clear that there must be some other type of interaction that helps to achieve a removal efficiency of 100% as some authors have already pointed out [39]. When the adsorbent is Fe_3_O_4_@Ag@L-Cys at a pH equal to 7, the removal efficiency would be expected to decrease; however, it remains at 100%, due to the lone pair of electrons in the sulfur atom of the cysteine, which can lead to a π=π interaction between these and the C=C double bonds of the PS, as has also been described by other authors [40].

### 2.5. Thermodynamic Studies of the Process

For the three adsorbents assayed, the isotherms corresponding to 273, 303, 323, and 343 K coincided since in all cases 100% removal efficiency was achieved for all the PS-MP concentrations studied. Only when the adsorbent was Fe_3_O_4_@Ag@L-Cys did the removal efficiency at 323 and 343 K slightly decrease and the corresponding isotherms could not be calculated. 

However, the Gibbs free energy for the optimal experimental conditions for the elimination of PS-MPs on each adsorbent can be calculated.

ΔG^0^ (kJ mol^−1^) is determined from the following equation:

∆G^0^ = −RTln (Kt) (1)

where T is the absolute temperature, R is the gas constant, and K_t_ is the equilibrium thermodynamic constant, whose value should be calculated from:

(2) $$K_{t}=\frac{\mathrm{qe}}{\mathrm{Ce}}$$

where qe (mg g^−1^) is the adsorption capacity and Ce (mg L^−1^) the adsorbate concentration.

The value of the standard Gibbs free energy ΔG^0^ (kJ mol^−1^) is indicative of whether the adsorption process corresponds to chemisorption, physisorption, or a combination of both [41]. Physisorption corresponds to the interval [ −20, 0] kJ mol^−1^ and chemisorption is within [−400, −80] kJ mol^−1^. The range in between can be labelled as a physicochemical adsorption [38]. The calculation of values of ΔG^0^ for the optimal and most favorable experimental conditions for each adsorbent, led to the following results.

When the adsorbent was Fe_3_O_4_, 100% removal of PS (50 mg L^−1^) was achieved at room temperature using 1.58 mg of adsorbent. In this case the ΔG^0^ calculated was −18.26 kJ mol^−1^. This result shows a physisorption process.

When the adsorbent was Fe_3_O_4_@Ag, total removal of PS (50 mg L^−1^) was achieved at room temperature using 2.16 mg of adsorbent and neutral pH, with ΔG^0^ equal to −17.6 kJ mol^−1^. Therefore, this case is also a physisorption process.

For Fe_3_O_4_@Ag@L-Cys, 100% removal of PS was achieved at room temperature, neutral pH with 3.01 mg of the adsorbent. The resulting ΔG^0^ was equal to −2.73 kJ mol^−1^ indicating a physisorption process again.

### 2.6. Study of the Recovery and Reuse of the Adsorbent and Application to Real Samples

In order to reuse the adsorbents, recycling studies were carried out to remove the PS-MPs from their surface and then reuse the sorbents for further removal of plastic from water.

After the removal of PS-MPs from water with each of the adsorbents studied in this work (Fe_3_O_4_, Fe_3_O_4_@Ag, and Fe_3_O_4_@Ag@L-Cys), these were removed from the water with a magnet, which after was placed in a conical tube. Then, 1 mL of a 0.1 M nitric acid solution was added. Ultrasound was then applied for different times (from 5 to 30 min). It was observed that with all adsorbents, after the application of 20 min of ultrasound, the PS-MPs particles were released from the surface of the sorbent material. After placing the magnet again at the bottom of the tube, the solution was decanted containing the PS, which was recycled.

The adsorbents were washed with three 5-milliter water aliquots and used again. It was found that after five reuse cycles they did not show any alteration in their adsorbent properties on testing the removal efficiency.

Moreover, to demonstrate that the proposed procedure can be applied to real water samples, three samples of both wastewater and tap water were fortified with 50 mg L^−1^ of PS-MPs. The proposed procedure for the removal of this pollutant using Fe_3_O_4_@Ag@L-Cys as adsorbent was applied to the fortified water samples. The results showed that in all cases 100% removal efficiency was achieved.

## 3. Materials and Methods

### 3.1. Materials

FeCl_2_·4H_2_O and FeCl_3_·6H_2_O obtained from Merck (Darmstadt, Germany) were used to synthesize iron oxide particles Fe_3_O_4_, which were the core for every adsorbent employed in this study. Concentrated ammonia (NH_3_) was supplied by Panreac Química SLU (Barcelona, Spain), NaBH_4_ was obtained from Fluka Chemicals (Buchs, Switzerland), and silver nitrate and L-Cysteine were both purchased from Sigma-Aldrich (St. Louis, MO, USA).

PS-MPs (849.57 ± 99.19 μm diameter) used as standards were purchased from GoodFellow GmbH (Hamburg, Germany). 

A vertical rotary carousel shaker, model MX-RD-E, and a thermostatic bath supplied by TierraTech (Cantabria, Spain) were used.

A Nikon D60 camera (Tokyo, Japan) and ImageJ software v1.53t were used for counting the MPs.

### 3.2. Preparation of Core Fe_3_O_4_ Nanoparticles

FeCl_2_·4H_2_O (0.2 g) and 0.56 g of FeCl_3_·6H_2_O were weighed and dissolved in 20 mL of ultrapure water previously heated to 80 °C in a thermostatic bath. 

Then, 2 mL of ammonium solution was added to the mixture drop by drop. The resulting brown suspension was stirred for 10 min using an orbital shaker. After that, the ferrite nanoparticles formed (Fe_3_O_4_) were separated from the aqueous solution using a neodymium magnet and the supernatant was removed. Fe_3_O_4_ NPs were finally washed several times with small portions of water and suspended in 20 mL of ultrapure water. The formation of Fe_3_O_4_ corresponds to the following chemical reaction [42]:

Fe^+2^ + 2Fe^+3^ + 8OH^−^ → Fe_3_O_4_ + 4H_2_O

### 3.3. Preparation of Fe_3_O_4_ Modified with Silver Nanoparticles (Fe_3_O_4_@Ag)

A AgNO_3_ solution (5.7 mL) at a concentration of 6.4·10^−5^mol L^−1^ was added to a previously synthesized Fe_3_O_4_ suspension (20 mL). The mixture was stirred for 5 min. Then, 14 mL of 3.2·10^−4^mol L^−1^ NaBH_4_ solution was slowly added, keeping the mixture agitated for an additional 10 min.

### 3.4. Preparation of Fe_3_O_4_ Modified with Silver Nanoparticles and Functionalized with L-cysteine (Fe_3_O_4_@Ag@L-Cys)

To 20 mL of a previously synthesized Fe_3_O_4_@Ag suspension, 2 mL of a solution of L-cysteine at a concentration of 0.1 mol L^−1^ was slowly added. Then, the mixture was kept under continuous agitation for 24 h.

### 3.5. Proposed General Process for the Removal of PS-MPs in Water Using Fe_3_O_4_, Fe_3_O_4_@Ag, and Fe_3_O_4_@Ag@L-Cys as Sorbents

An aqueous solution containing PS-MPs microspheres at a concentration of 50 mg L^−1^ was treated with the three adsorbents studied, obtaining in all cases a total elimination of the plastic microspheres. Every study described above in this paper was carried out in triplicate.

When the adsorbent used was Fe_3_O_4_, it was first necessary to heat the aqueous solution to 50 °C, then add 100 µL of the adsorbent suspension and keep it stirred for 15 min. After this time, the neodymium magnet was added, and the aqueous solution decanted totally free of PS-MPs.

When the composite used was Fe_3_O_4_@Ag, 250 µL of the material suspension and 30 min of continuous agitation were necessary to achieve 100% elimination efficiency of polystyrene in water. However, it was not necessary to heat the initial water solution.

When Fe_3_O_4_@Ag@L-Cys was employed as adsorbent, the total elimination of PS-MPs was reached using 50 μL of sorbent suspension, after 15 min under continuous agitation at room temperature.

### 3.6. Calculations of Removal Rate of MPs

In the experiments for removing PS-MPs from water, the removal rate was measured according to the number of MPs (counting). Following this equation, ϴ is the removal rate of PS-MPs (%), n is the number of remaining PS-MPs in the water, and N is the number of total initial added PS-MPs in the sample (N is 2 or 3):

ϴ = 100 − (n/N × 100)(3)

### 3.7. Procedure for Counting MPs

The treated water sample was filtered with a 25 µm metallic filter to calculate the remaining MPs in the water and, therefore, evaluate the removal efficiency. The filters were left in an oven overnight at 60 °C to be dried. Then, a Nikon D60 camera with optical zoom at 1x was used to take pictures of the filter with the remaining PS-MPs. Manual photos from the different samples were processed by ImageJ v1.53t software providing the number of PS-MPs present. PS-MPs used in this study were spherical with an average size of 849.57 ± 99.19 µm, making this analysis straightforward for the detection of these types of particle. In addition, potential MPs contamination would be detected and avoided due to the different shape and size.

## 4. Conclusions

Microplastics are emerging pollutants that are currently of concern to the scientific community and the public because of the consequences of their presence in the trophic chain. The development of new methods for the elimination of these pollutants in water is a common goal of many researchers. 

A comparative study has been developed for the removal of PS-MPs using three magnetic adsorbents, namely Fe_3_O_4_, Fe_3_O_4_@Ag, and Fe_3_O_4_@Ag@L-Cys. Different experimental conditions have been studied for the elimination of 50 mg L^¯1^ of polystyrene microspheres using each adsorbent, specifically, temperature, contact time, pH, and adsorbent dose. The optimal conditions to achieve the total removal of PS-MPs in water with each sorbent have been proposed. In addition, a removal mechanism has been suggested for each of them. Comparative results showed that the complete elimination of PS-MPs in water samples was achieved with Fe_3_O_4_@Ag@L-Cys within 15 min at room temperature and neutral pH. In addition, when the adsorbent used was Fe_3_O_4_@Ag@L-Cys the volume of adsorbent suspension needed to totally remove the PS-MPs was much lower than in the other two cases, needing only 50 µL. Given the short time, adsorbent dosage, and proposed removal mechanism, which seems more robust than with the other adsorbents tested, the authors consider Fe_3_O_4_@Ag@L-Cys to be the most suitable adsorbent to remove polystyrene microspheres in water. However, the good results also achieved using Fe_3_O_4_, which only needs 30 min of contact time and 1.58 mg of this material to reach total elimination of the PS-MPs, make it a good alternative when there is no possibility to use Fe_3_O_4_@Ag@L-Cys in the same way. 

The thermodynamic study of the processes for each adsorbent showed that the removal of PS-MPs occurs spontaneously and that it is a physisorption process. In addition, recovery and recycling studies of the adsorbents were carried out successfully, managing to recover each one by applying a few minutes of ultrasound in an acidic medium. The three adsorbents were used for further PS-MPs elimination without losing their adsorbent capacities.

Therefore, in this work, a fast and efficient strategy allowed the total elimination of PS-MPs from an aquatic environment using Fe_3_O_4_@Ag@L-Cys as adsorbent, which was synthesized in a simple and low-cost way. This procedure may be considered for application to real samples, aiding the elimination of MPs in a faster and low energy cost methodology bringing an alternative solution to current contamination challenges. 

## Figures and Tables

**Table 1:** Dependence of the contact time between the adsorbent and the aqueous solution on the adsorption efficiency.

Adsorbents	Removal Efficiency at Different Times (%)			
	5 min	15 min	30 min	60 min
Fe_3_O_4_	50%	70%	100%	100%
Fe_3_O_4_@Ag	50%	50%	100%	100%
Fe_3_O_4_@Ag@L-Cys	50%	100%	100%	100%

## Data Availability

Data are contained within the article.
